# Tumour Cells Expressing Single VEGF Isoforms Display Distinct Growth, Survival and Migration Characteristics

**DOI:** 10.1371/journal.pone.0104015

**Published:** 2014-08-13

**Authors:** Chryso Kanthou, Gabi U. Dachs, Diane V. Lefley, Andrew J. Steele, Claudia Coralli-Foxon, Sheila Harris, Olga Greco, Sofia A. Dos Santos, Constantino C. Reyes-Aldasoro, William R. English, Gillian M. Tozer

**Affiliations:** 1 Tumour Microcirculation Group, CR-UK/YCR Sheffield Cancer Research Centre, The University of Sheffield, Department of Oncology, School of Medicine, Sheffield, United Kingdom; 2 Mackenzie Cancer Research Group, Department of Pathology, University of Otago, Christchurch, New Zealand; 3 Cancer Sciences Unit, Faculty of Medicine, University of Southampton, Southampton, United Kingdom; 4 University of Sheffield, Sheffield, United Kingdom; Ottawa Hospital Research Institute, Canada

## Abstract

Vascular endothelial growth factor-A (VEGF) is produced by most cancer cells as multiple isoforms, which display distinct biological activities. VEGF plays an undisputed role in tumour growth, vascularisation and metastasis; nevertheless the functions of individual isoforms in these processes remain poorly understood. We investigated the effects of three main murine isoforms (VEGF188, 164 and 120) on tumour cell behaviour, using a panel of fibrosarcoma cells we developed that express them individually under endogenous promoter control. Fibrosarcomas expressing only VEGF188 (fs188) or wild type controls (fswt) were typically mesenchymal, formed ruffles and displayed strong matrix-binding activity. VEGF164- and VEGF120-producing cells (fs164 and fs120 respectively) were less typically mesenchymal, lacked ruffles but formed abundant cell-cell contacts. On 3D collagen, fs188 cells remained mesenchymal while fs164 and fs120 cells adopted rounded/amoeboid and a mix of rounded and elongated morphologies respectively. Consistent with their mesenchymal characteristics, fs188 cells migrated significantly faster than fs164 or fs120 cells on 2D surfaces while contractility inhibitors accelerated fs164 and fs120 cell migration. VEGF164/VEGF120 expression correlated with faster proliferation rates and lower levels of spontaneous apoptosis than VEGF188 expression. Nevertheless, VEGF188 was associated with constitutively active/phosphorylated AKT, ERK1/2 and Stat3 proteins. Differences in proliferation rates and apoptosis could be explained by defective signalling downstream of pAKT to FOXO and GSK3 in fs188 and fswt cells, which also correlated with p27/p21 cyclin-dependent kinase inhibitor over-expression. All cells expressed tyrosine kinase VEGF receptors, but these were not active/activatable suggesting that inherent differences between the cell lines are governed by endogenous VEGF isoform expression through complex interactions that are independent of tyrosine kinase receptor activation. VEGF isoforms are emerging as potential biomarkers for anti-VEGF therapies. Our results reveal novel roles of individual isoforms associated with cancer growth and metastasis and highlight the importance of understanding their diverse actions.

## Introduction

Vascular endothelial growth factor-A (VEGF) plays a fundamental role in tumour growth, vascularisation and metastasis and exists as multiple isoforms derived by alternative splicing of the VEGF gene [1]. Mouse and human proteins of 120/121, 164/165 and 188/189 amino acids respectively, represent major VEGF splice variants with distinct properties and expression patterns. These isoforms differ in terms of binding affinities to the extracellular matrix and receptor activation. Tumours display highly variable levels of relative isoform expression, with VEGF-164/165 and VEGF120/121 generally being the most predominant and VEGF-188/189 relatively less abundant [2]. VEGF signals through tyrosine kinase receptors VEGFR1/flt-1, VEGFR2/flk-1 and VEGF3/flt-4 [3]. VEGF also binds neuropilin co-receptors (NRP-1 and NRP-2), which lack tyrosine kinase activity but regulate the function of VEGF receptors as well as other receptor tyrosine kinases (RTKs) [3]. The different affinities to matrix, displayed by the various VEGF splice variants generate gradients *in vivo* and result in different signalling responses, which are important for angiogenesis [4], [5]. VEGF also has complex functions in angiogenesis-independent aspects of tumour growth and tumour cells have been shown to express functional VEGF receptors [6], [7], [8] but the role of individual VEGF isoforms in these processes remains poorly understood.

VEGF and its receptors are now major targets of several cancer therapies. Anti-VEGF agents such as the humanised neutralising anti-VEGF antibody bevacizumab as well as several VEGF receptor kinase inhibitors are being used to treat many types of cancer. However, not all patients respond to anti-VEGF therapy and therefore biomarkers that can predict clinical response are being actively pursued [9]. Indeed, several recent retrospective clinical studies have identified the short soluble isoforms of VEGF (VEGF120 and 110) as promising potential biomarkers for predicting responsiveness to bevacizumab [10], [11], [12]. Pancreatic, breast and gastric cancer patients with higher circulating levels of short VEGF121 and VEGF110 isoforms were shown to have a shorter median overall survival but were more likely to benefit from treatment with bevacizumab. Importantly, the short VEGF isoforms were identified out of a comprehensive range of potential biomarker candidates that were examined in these studies [8], [9], [10]. These studies highlight the importance of understanding of the complexities associated with the functions of individual VEGF isoforms in cancer.

Immortalization and transformation of mouse embryonic fibroblasts results in cell lines with tumorigenic potential. Such cells derived from genetically modified animals are useful for studying the role of specific genes in cancer progression [13]. Using this approach we developed VEGF isoform-specific fibrosarcomas [14] by H-rasV12 transformation of embryonic fibroblasts derived from transgenic animals expressing single VEGF isoforms and wild type controls [15], [16]. These cells produced tumours *in vivo* that displayed distinct vascular patterns, similar to those seen in corresponding transgenic animals during development. Furthermore, the tumours displayed differences in response to treatment with vascular targeting agents highlighting the importance of VEGF isoform expression in treatment outcome [14], [17]. When grown *in vitro*, the fibrosarcoma cells exhibited major differences in growth rates, with VEGF164 and VEGF120 expressing cells (fs164 and fs120 respectively) proliferating significantly faster than VEGF188 (fs188) and wild type control cells (fswt) [14] suggesting that VEGF isoform expression also controls tumour cell growth characteristics. Initial growth rates were also faster for fs164 and fs120 tumours *in vivo*, which are consistent with the highly proliferative phenotype of the cells [14].

Proliferation and survival of cancer cells is maintained by constitutive activation of multiple complex signalling pathways that ordinarily maintain tight control in normal cells [18]. Mutated proto-oncogenes such as Ras can cause aberrant signalling through the Raf/MEK/ERK1/2 and PI3K/AKT signalling cascades that regulate tumour cell growth and survival. Multiple signalling mechanisms also converge to co-ordinate tumour cell invasiveness and migration. In particular, assembly of the actin cytoskeleton, under the control of the Rho family of GTPases, is required so that protrusions and contractile forces can be generated to propagate cell movement [19]. Cancer cells move either individually or collectively as multicellular clusters and maintain the capacity to switch from one mode of movement to another. Individual cell migration is classified as either “mesenchymal” or “amoeboid” and each mode has different requirements for contractility, attachment to matrix and proteolysis [20].

VEGF is known to contribute to intracrine and autocrine tumour cell growth, survival, migration and invasion [7], [8]. We hypothesise that individual isoforms of VEGF might influence various aspects of tumour cell behaviour in a distinct manner. Using the fibrosarcoma cells we developed, which express single VEGF isoforms and, in parallel, wild type control fibrosarcoma cells capable of expressing all isoforms, we investigate here the consequences of endogenous isoform expression on tumour cell morphology, proliferation, survival and migration. Using this system, in which VEGF expression is maintained under the control of its endogenous physiological promoter, we have discovered that individual isoforms are associated with distinct roles in terms of tumour cell behaviour and gained insight into signalling pathways that are linked with these effects.

## Materials and Methods

### Ethics statement

Animal experiments were conducted according to United Kingdom Animals (Scientific Procedures) Act 1986 (UK Home Office Project Licence PPL40/3110) and with local University of Sheffield ethical approval.

### Reagents

Blebbistatin, UO126, Y27632 and pyridone 6 (P6) were purchased from Calbiochem. SU11248 (sunitinib) was purchased from LC laboratories. Antibodies to integrin β1, ILK, pAKT (ser473), p70S6 (thr389), pGSK-3 (ser21/9), pFOXO-1 (ser256), p-Stat3 (tyr705), VEGFR2, pVEGFR2 (tyr996), pVEGFR2 (tyr1175) and GAPDH were from Cell Signalling. Anti- actin, pERK, tERK and β-tubulin antibodies were from Sigma. N-cadherin, p21 and p27 antibodies were from BD Biosciences and LIF antibody was from R&D Systems. VEGFR1 (ab32152 and ab2350) and pVEGFR1 (*tyr1333*) antibodies were from Abcam. Recombinant VEGF164, VEGF165 and VEGF120 proteins were purchased from Peprotech. Recombinant VEGF188 protein was purchased from Reliatech GmbH.

### Cell culture

Fibrosarcoma cell lines (fs188, fs164, fs120 and fswt) were developed as we described before [14], from fibroblasts derived from transgenic mouse embryos expressing single VEGF isoforms VEGF188, VEGF164, VEGF120 and wild type controls respectively [15], [16]. Cells were maintained in Dulbecco's modified Eagle's medium (DMEM), 10% FCS, 600 µg/ml G418 and 2 µg/ml puromycin (Invitrogen, UK). The mouse endothelial H5V cell line was a gift from Dr Annunciata Vecchi [21]. H5V cells were maintained in DMEM containing 10% FCS. Human umbilical vein endothelial cells (HUVEC) from pooled donors were purchased from PromoCell and were maintained in endothelial cell growth medium (PromoCell).

### Preparation of conditioned media

Cells were grown to 50% confluence and then switched to serum-free DMEM for 48 h. Conditioned media were concentrated 30-fold with Microcon concentrators (Merck Millipore) and normalised to cell numbers before they were analysed by immunoblotting.

### Preparation of collagen, fibronectin and laminin matrices

A thin coating of monomeric collagen was prepared by incubating culture plates for 2 h with 50 µg/ml rat tail collagen type I (BD Biosciences) diluted in 0.02 N acetic acid. Bovine fibronectin (Invitrogen, UK) or laminin-1 (Sigma) were diluted to 5 µg/ml in PBS and used to coat dishes for 2 h. To prepare a thick layer of fibrillar matrix, ice-cold rat-tail type I collagen was diluted to 1.5 mg/ml with 10XDMEM and sterile dH_2_O. The solution was neutralised with NaOH and allowed to polymerise at 37°C before plating the cells on top.

### Adhesion and cell spreading assays

Adhesion assays were performed in 96-well plates pre-coated with fibronectin, laminin or collagen and blocked in 0.1% BSA. Cells (3×10^4^) suspended in serum-free DMEM/0.1% BSA were plated in each well, and plates were immediately incubated at 37°C for 45 min. Adherent cells were fixed, stained with 0.5% crystal violet and lysed in 2% SDS. Absorbance was measured at 570 nm using a BMG FLUORStar Galaxy microplate reader. For monitoring spreading, cells were re-suspended in DMEM/0.1% BSA and plated in Lab-Tek 4-well permanox chamber slides (Nalge-Nunc) (1×10^5^ per well) pre-coated with collagen, laminin or fibronectin. The slides were incubated at 37°C for 30 min to 3 h and then cells were stained as described below.

### Analysis of cell morphology and cytoskeletal F-actin staining

Live cells were stained with 2.5 µg/ml CellMask plasma membrane stain (Invitrogen, UK) for 5 min, fixed in 3.7% formalin and mounted in Vectashield (Vector Laboratories). Analysis of F-actin was performed as we described previously [22]. Fluorescence images were taken with a Leica DMI4000B fluorescence microscope and using LASAF control and analysis software.

### Growth curves and doubling times

Cells were plated in 6-well plates (4×10^4^ per well). Viable cells were counted daily for 4–6 days using a Vi-Cell automated cell viability analyzer (Beckman Coulter). Media were replenished every other day. Doubling times were established by plotting a graph of log 2 (N/N0) (N0 = initial cell numbers and N = end-point average cell numbers) against time followed by calculating the inverse slope of the linear part of the curve.

### Colony formation in soft agar

Cells were suspended in medium containing 0.33% agarose and plated over a 0.5% agarose underlay in 6-well plates (2×10^3^ cells/well). Three weeks later, colonies were stained with 0.005% crystal violet and counted in 10 random fields using a 4× objective.

### Analysis of apoptosis

Apoptotic cell death was measured by flow cytometry using the ApoAlert MitoSensor Kit (Clontec) which detects alterations in mitochondrial membrane potential. Apoptosis was also quantified using the Cell Death Detection ELISA^PLUS^ kit (Roche Diagnositics), which measures cytoplasmic DNA-histone nucleosome complexes. For both assays cells were plated at a density of 10^4^ cells/cm^2^ and apoptosis was determined 48 h later. Apoptosis in tumour sections was detected by TUNEL assay using the ApoTag Plus Peroxidase *In Situ* Apoptosis Detection kit (Chemicon International). Apoptotic cells were scored using a 40× objective in 6–9 random fields of view per tumour section avoiding areas of necrosis. Data are expressed as mean number of TUNEL positive apoptotic cells per mm^2^.

### Migration assay

Migration was measured using Ibidi cell culture inserts. Cells (3.5×10^4^) in 70 µl medium were plated into each insert compartment and grown for 48 h. The inserts were removed to create a gap/wound of 500 µm into which the cells migrated. The cells were imaged immediately and at intervals for up to 24 h with a Nikon Eclipse phase contrast microscope equipped with a Digital Slight DS camera and NIS-Elements software. Wound closure was quantified with an automatic image analysis algorithm [23] using the CAIMAN image analysis website (http://www.caiman.org.uk/).

### Subcutaneous tumour generation

Fibrosarcoma cells (1×10^6^ in 50 µl serum-free DMEM) were injected subcutaneously into the rear dorsum of female 8–12 week-old SCID mice. Tumours were excised when they reached a mean diameter of 6–8 mm (usually 10–14 days post-implantation) and were either embedded in paraffin and sectioned for analysis of apoptosis, or were frozen for protein extraction.

### Immunoblotting

Proteins from cells and tumours were extracted with NP40 Lysis Buffer (Biosource, Invitrogen) supplemented with phosphatase and protease inhibitors. Equal amounts of protein (10–50 µg/lane) were separated on NuPAGE Novex gels (Invitrogen), transferred to nitrocellulose or PVDF membranes and immunoreactive bands were visualized using ECL reagents (GE Healthcare).

### Statistical analysis

Data were analysed by using a one-way analysis of variance (ANOVA), followed by the Tukey-Kramer post-test test for multiple comparisons using GraphPad Prism software for Mac OS X. A 2-way ANOVA followed by a Bonferroni post-test was used for analyzing multiple groups with more than one variable (treatment and cell type). In all cases, differences between groups were described as significant if the probability was <0.05.

## Results

### Fibrosarcoma cells expressing individual VEGF isoforms differ in morphology and adhesion properties

**Figure 1:**
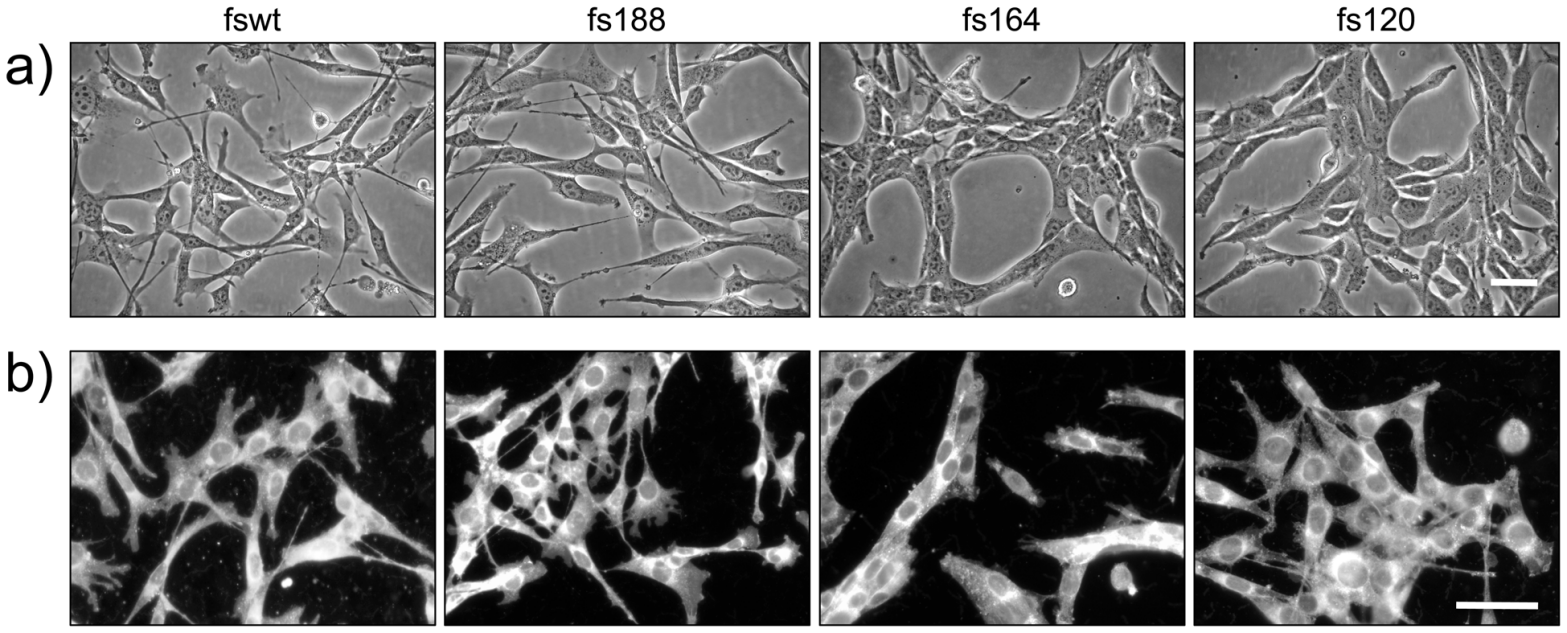
Morphology of fibrosarcoma cells expressing single VEGF isoforms. **a**): Phase contrast images of cells grown on uncoated tissue culture plastic. **b**): Cells grown on plastic and stained live with CellMask Orange. Scale bars, 50 µm.

**Figure 2:**
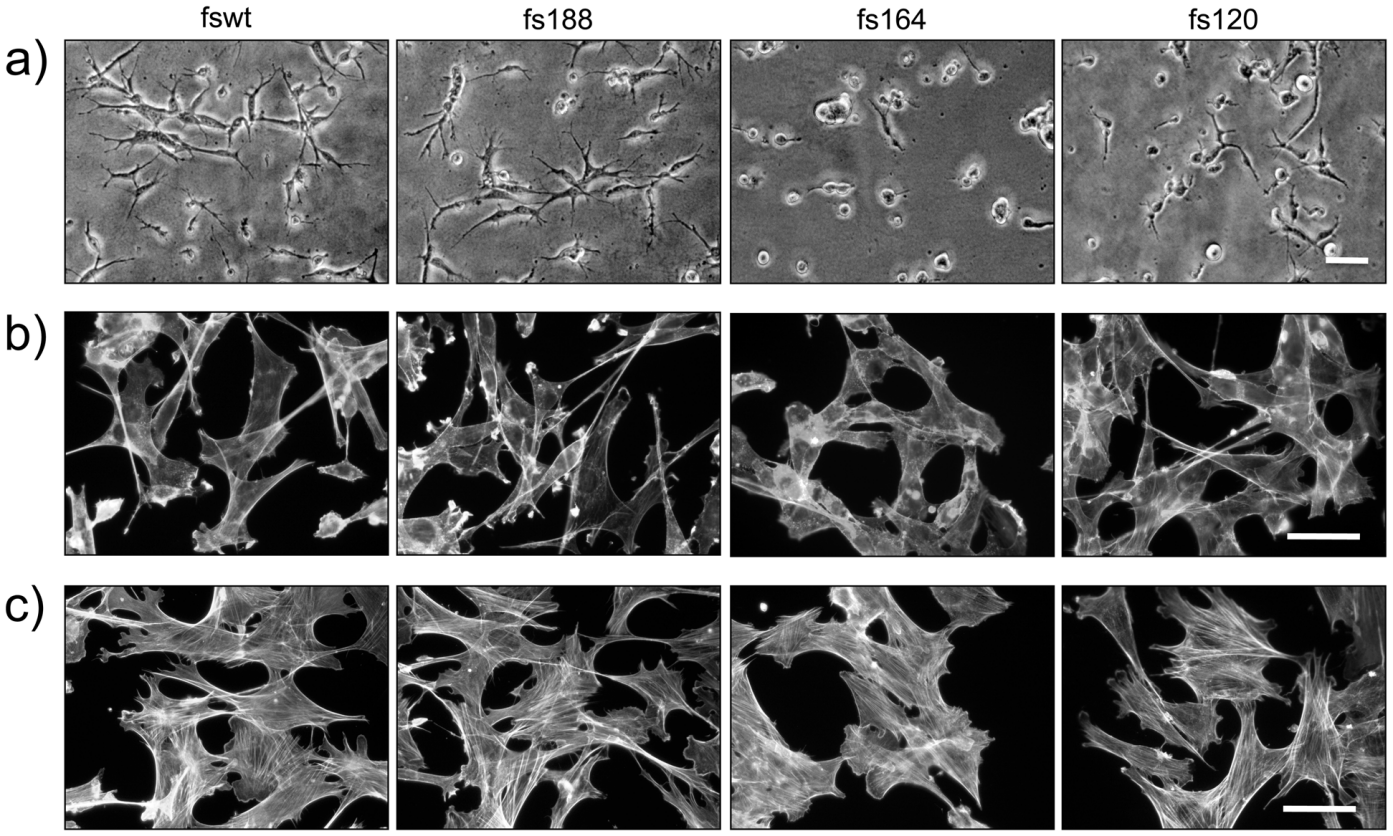
Morphology of fibrosarcoma cells on thick fibrillar collagen and after inhibition of MEK. **a**): Cells on a thick layer of type I collagen; **b**): cells grown on uncoated plastic and stained for F-actin. **c**): Cells treated with MEK inhibitor U0126 (10 µM) for 18 h and stained for F-actin. Scale bars, 50 µm.

Fs188 as well as fswt cells, which express all isoforms including VEGF188, display typical mesenchymal features (Figure 1a,b). The cells are elongated and spindle-shaped, have extended processes and ruffles and do not form extensive cell-cell associations. In contrast, fs164 do not display a typical mesenchymal morphology. These cells grow in very close association with each other and closely align longitudinally forming long multicellular chains. Fs120 cells also grow in close association with each other, although less so than fs164 cells, and also have fewer extended processes than fs188 and fswt cells. On fibrillar collagen, fswt and fs188 cells retained their mesenchymal features while fs164 cells were rounded/amoeboid and fs120 cells displayed a mix of rounded and elongated features (Figure 2a). All fibrosarcoma cells stained diffusely for F-actin (Figure 2b), a characteristic feature of Ras transformation, which elevates ERK MAPK signalling and uncouples Rho-GTP signalling from stress fibre formation [24], [25]. Inhibition of ERK activator MEK with U0126 reversed the effects of transformation on F-actin, and re-established stress fibers in all the cell lines (Figure 2c). In addition, fs120 cells assumed a more mesenchymal morphology and displayed more extended processes. Fs164 cells expressed stress fibres upon MEK inhibition although their distinct close cell-cell alignment was still evident, suggesting that this feature was not MEK-dependent.

**Figure 3:**
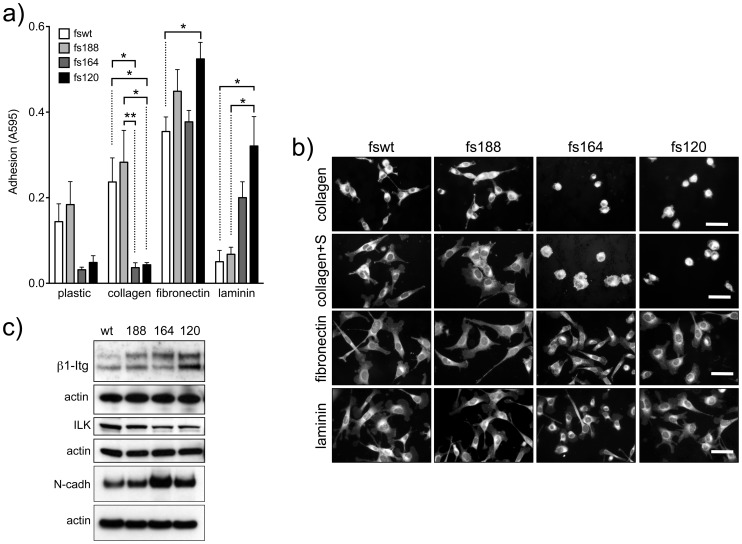
Fibrosarcoma cell adhesion and spreading. **a**): Cell adhesion to uncoated plastic or plastic coated with collagen I, fibronectin or laminin-1 at 45 min. * and ** represent significant differences, (*p<0.05 and **p<0.01) by two way ANOVA followed by Bonferroni post-test. **b**): Cell spreading on fibronectin and laminin at 30 min, on a thin layer of collagen at 3 h or on collagen for 3 h in the presence of 2% FCS (collagen+S). Cells were stained live with with CellMask orange. Scale bars, 50 µm. **c**): Immunoblot analysis for β1 integrin (β-Itg) ILK and N-cadherin. Blots were normalised to actin. Results are representative of at least 3 independent experiments.

Fswt and fs188 cells adhered better to uncoated plastic and collagen than fs164 and fs120 cells (Figure 3a). On the other hand, fs164 and fs120 cells adhered better to laminin than fswt and fs188 cells. All four cell lines adhered to and also spread avidly and rapidly on fibronectin (Figure 3a, b). All cells also spread on laminin but fs164 and fs120 cells failed to spread on collagen, even after 3 h and in the presence of serum (Figure 3b). All the fibrosarcoma cells expressed integrin β1 and integrin-linked kinase (ILK) (Figure 3c), an adaptor protein that is recruited to integrin β1 cytoplasmic domains and associates with actin at focal adhesions [26]. ILK expression levels were higher in fswt and fs188 compared to fs164 and fs120 cells. All fibrosarcomas also expressed N-cadherin (Figure 3c), a classic mesenchymal marker [27]. Levels of N-cadherin were up-regulated in fs164 and fs120 cells, which also formed more cell-cell contacts (also see Figure 1).

### Fibrosarcoma cells expressing individual VEGF isoforms proliferate at different rates and display differences in levels of spontaneous apoptosis

**Figure 4:**
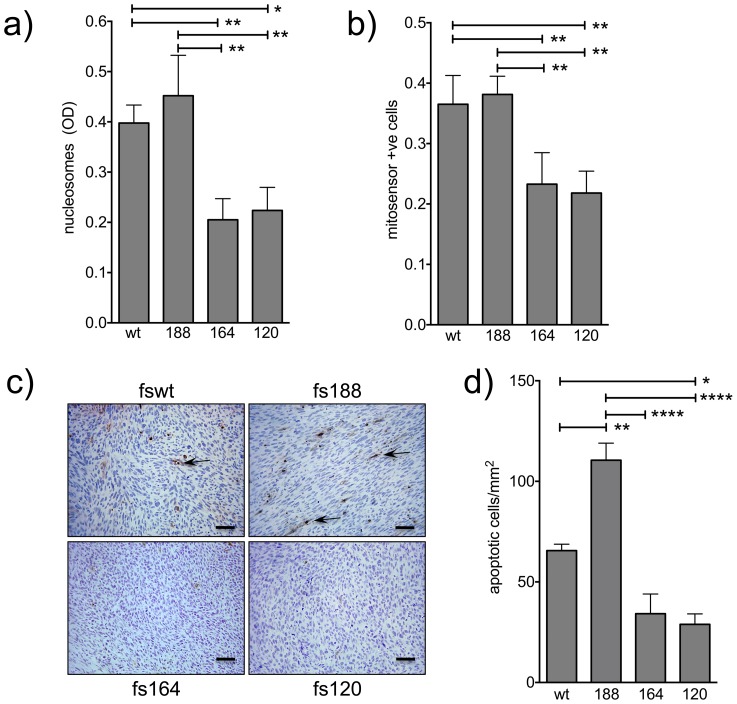
Spontaneous apoptosis in fibrosarcoma cells and solid tumours. **a**): Nucleosome generation in cells measured by ELISA at 48 h. OD values are averages of 5 independent experiments ± SEM. **b**): Changes in cell mitochondrial potential at 48 h. Values are averages ± SEM, from 4 independent experiments. *p<0.05, **p<0.01 represent significance using repeated measures ANOVA followed by Tukey-Kramer post-test. **c**): Apoptosis in tumour sections determined by TUNEL assay. Arrows indicate typical apoptotic cell nuclei. Bars, 50 µm. d): Mean number of TUNEL positive apoptotic cells ± SEM per mm^2^ tumour section (viable regions only); n = 5–10 tumours per group. *p<0.05, **p<0.01 and ****p<0.0001 represent significance using repeated measures ANOVA followed by Tukey-Kramer post-test.

We previously showed that fs164 and fs120 cells proliferated on plastic at significantly faster rates than fswt and fs188 cells [14]. Integrin-matrix interactions play an important role in controlling cell proliferation. However, neither collagen nor fibronectin had any significant effect on growth, and the differences in proliferation between the cell lines persisted (Table I). Growth in soft agar, an indication of tumourigenic capacity, was also significantly different between the cell lines; fs164 and fs120 cells producing significantly more colonies than fswt and fs188 cells (Table II and also see Figure S1). Apoptosis was significantly more pronounced in fswt and fs188 cells than in fs164 and fs120 cells (Figure 4a,b). Similarly, *in vivo*, there were significantly more apoptotic cells within viable regions of fs188 solid tumours compared to tumours generated by fs164 and fs120 cells (Figure 4c,d).

**Table 1:** Population doubling times (hours) of fibrosarcoma cells grown on 2D surfaces.

	fswt	fs188	fs164	fs120
Plastic	18.36±0.29 (n = 6)	17.89±0.336 (n = 6)	***13.89±0.57 (n = 6)	***13.67±0.26 (n = 6)
Collagen	16.98±0.92 (n = 6)	17.17±0.24 (n = 3)	**13.15±0.09 (n = 3)	*14.49±0.46 (n = 3)
Fibronectin	19.41±1.175 (n = 6)	18.87±1.55 (n = 3)	***13.55±0.24 (n = 3)	***14.59±0.69 (n = 3)

Population doubling times (hours) of cells grown on plastic, collagen or fibronectin are means of 3–6 independent experiments ± SEM.

*p<0.05,

**p<0.01,

***p<0.001 values represent differences between fswt/fs188 cells versus fs164/fs120 cells (two way ANOVA followed by Bonferroni post-test).

**Table 2:** Colonies formed by fibrosarcoma cells grown in soft agar.

	fswt	fs188	fs164	fs120
Soft Agar	23.4±2.85 (n = 3)	24.98±4.4 (n = 3)	*33.8±3.7 (n = 3)	*35.8±4.2 (n = 3)

Number of colonies in agar are means of three independent experiments ± SEM.

*p<0.05 represents significantly more colonies formed by fs164 or fs120cells compared to fswt or fs188 cells (ANOVA followed by Tukey-Kramer post-test).

### Fibrosarcoma cells expressing individual VEGF isoforms migrate at different rates on 2D surfaces

**Figure 5:**
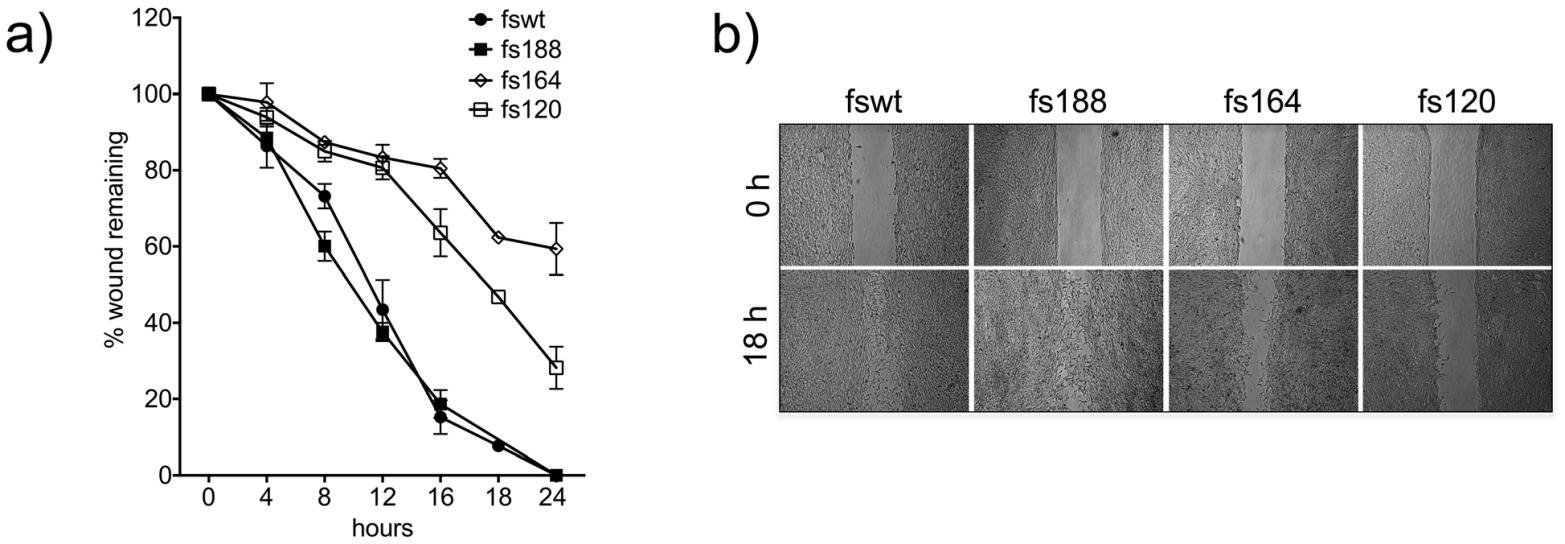
Migration of fibrosarcoma cells expressing single VEGF isoforms. **a**): Wound closure was measured at intervals up to 24 h. Each point represents data obtained from 2–5 independent experiments ± SEM; **b**): representative culture images of the four fibrosarcoma cell lines showing wound closure at 18 h.

Fswt and fs188 cells migrated significantly faster than fs164 and fs120 cells on 2D uncoated plastic (Figure 5). The faster migration of VEGF188-expressing cells was consistent with their mesenchymal morphology and presence of ruffles (see Figure 2b), an indication of intense migratory activity [28]. Migration rates on thin collagen were similar to migration rates on uncoated plastic (data not shown).

### Migration of fs164 and fs120 cells is actinomyosin contractility-dependent

**Figure 6:**
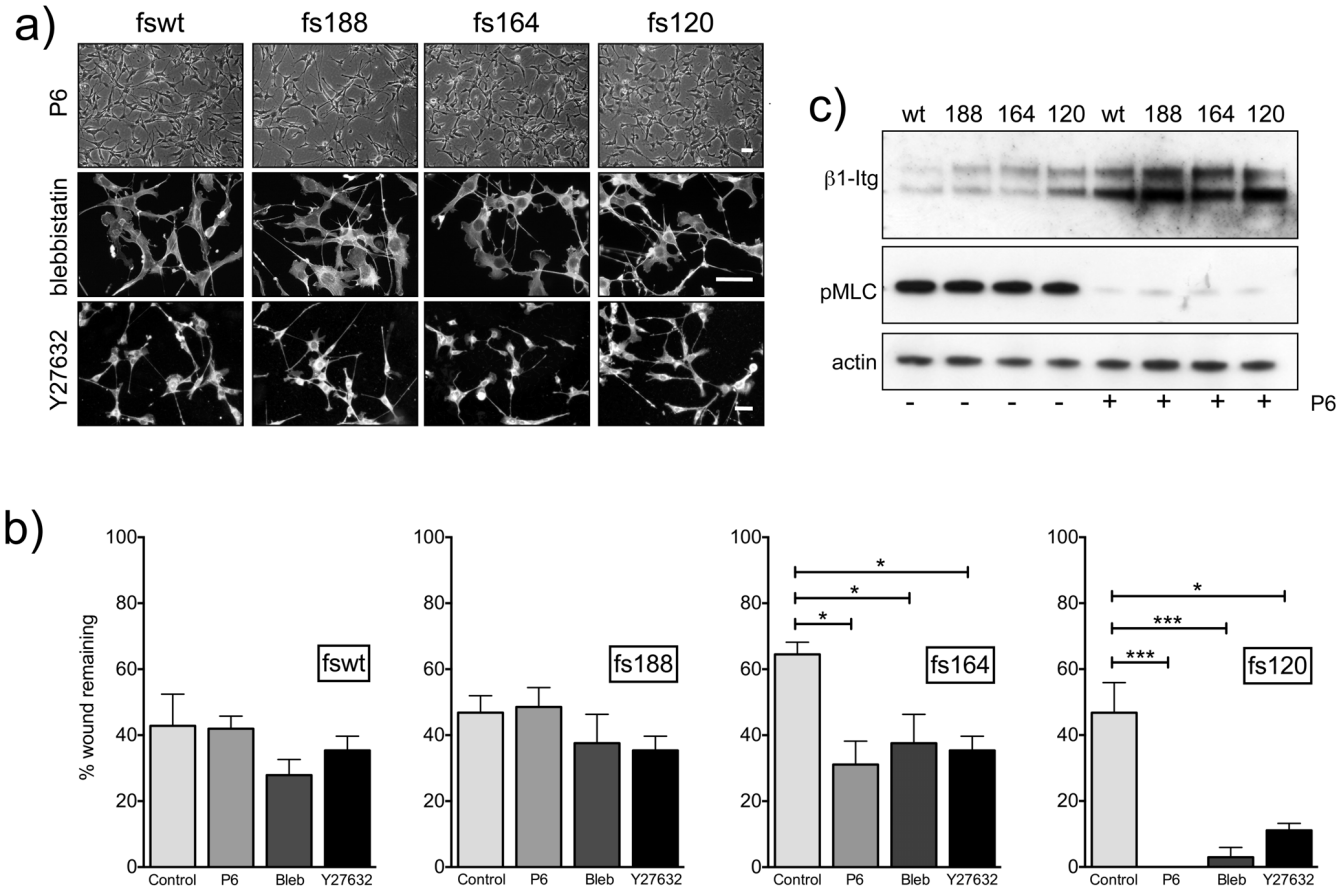
Analysis of morphology and migration of fibrosarcoma cells exposed to contractility inhibitors. **a**): Cells were treated with P6 (5 µM), blebbistatin (5 µM) or Y27632 (10 µM) for 18 h. Representative images of P6 treated cells by phase contrast microscopy; blebbistatin-treated cells stained for F-actin; Y27632 treated cells stained with CellMask Orange. Scale bars, 50 µm. **b**): Migration was quantified by measuring wound closure. Images were collected at 12 h for the fswt and fs188 cells, at 18 h for the fs120 cells and at 24 h for the fs164 cells to correlate with 40–60% wound closure in untreated conditions. Contractility inhibitors were added at the same concentrations used in (**a**) 18 h before the start of the assay and replaced in fresh media at assay start. Each point represents data obtained from 3 independent experiments ± SEM, each conducted with 2–4 replicates. *p<0.05, ***p<0.001 values represent significance over untreated cultures (ANOVA followed by Tukey-Kramer post-test). **c**): Cells were incubated overnight with P6 (5 µM). Equal amounts of proteins (30 µg) were analysed for integrin β1 (β-Itg) and pMLC. A representative immunoblot is shown.

Cell morphology and migration are dependent on the cytoskeleton and actinomyosin contractility, to provide traction force in mesenchymal movement, and cortical contraction for rounded amoeboid movement [19], [29]. Cells were treated with pharmacological inhibitors of contractility, including ROCK inhibitor Y27632, myosin II ATPase inhibitor blebbistatin and JAK inhibitor P6. The latter was shown to inhibit actinomyosin contractility in melanoma cells [30]. Figure 6a shows that all three inhibitors caused fs164 and fs120 cells to switch to a mesenchymal morphology (compare Figure 6a with Figure 1) and accelerated their migration but not that of fswt and fs188 cells (Figure 6b). P6 inhibited MLC phosphorylation in all the fibrosarcoma cells (Figure 6c) as previously shown for melanoma cells [30] but interestingly also induced a marked up-regulation of integrin β1 (Figure 6c).

### Fibrosarcoma cells expressing individual VEGF isoforms exhibit differences in growth and survival-associated signalling

**Figure 7:**
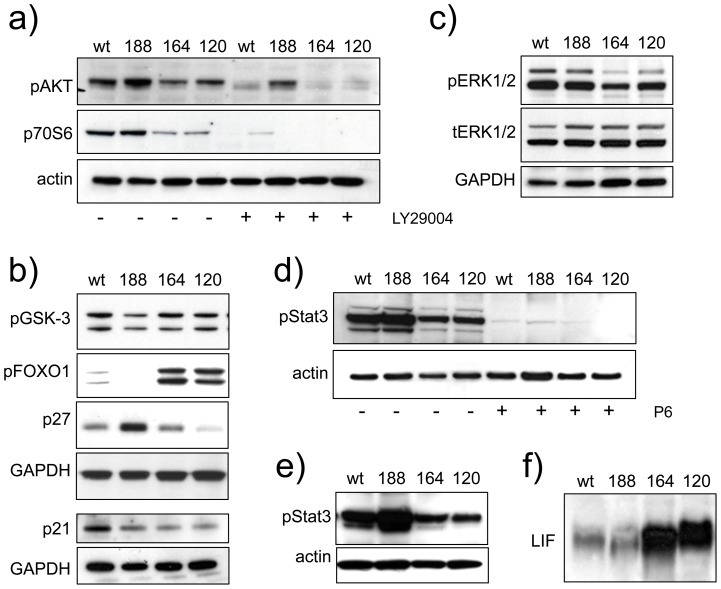
Signalling pathway analysis in fibrosarcomas expressing single VEGF isoforms. **a–d**): Where indicated cells were incubated overnight with JAK inhibitor P6 (5 µM), or PI3K inhibitor LY20990 (10 µM). Equal amounts of proteins (10–30 µg) were analysed for **a**): phospho-AKT (p-AKT) and p70S6; **b**): pGSK3, pFOXO1, p27 and p21; **c**): phospho-ERKs1/2 (p-ERK) and total ERK (t-ERK); **d**): phospho-Stat3 (p-Stat3). **e**): Proteins extracted from solid tumours (50 µg/lane) and analysed for pStat3. All blots were normalized with actin or GAPDH and are representative of at least three independent experiments. **f**): Concentrated conditioned media normalised against cell numbers, analysed for LIF expression.

We next investigated signalling mechanisms that could potentially account for the observed differences in cell growth, survival and migration. Signalling via AKT promotes growth through activation of the mTOR1 complex and downstream targets such as p70S6 kinase involved in cell growth and G1 cell cycle progression [18]. AKT and p70S6 were more highly phosphorylated in fswt and fs188 cells, compared to fs164 and fs120 cells (Figure 7a) and this was independent of cell density (data no shown). Overnight exposure to PI3K inhibitor LY29004 reduced constitutive phospho-AKT and p70S6 levels (Figure 7a). AKT also signals through FOXO and glycogen synthase kinase-3 (GSK-3) to regulate survival, metabolism and growth [31]. GSK-3 is active when unstimulated and becomes switched off upon phosphorylation by AKT. FOXO1 is also phosphorylated and inactivated by AKT and this action blocks its transcriptional activity on genes that promote apoptosis and regulate metabolism. Paradoxically, levels of pGSK-3 and pFOXO1 did not parallel AKT and p70S6 phosphorylation (Figure 7b). Higher levels of phosphorylated FOXO1 were detected in fs164 and fs120 cells in which basal pAKT was low, suggesting that AKT signalling was uncoupled from this downstream branch of the pathway. Similarly, pGSK-3 was not upregulated as might be expected in VEGF188-expressing cells. Both FOXO1 and GSK-3 mediate cell cycle arrest by regulating expression and stability of cyclin-dependent kinase inhibitors (CDKIs) [32], [33]. In fs188 cells, CDKI p27 was significantly upregulated, while in fswt cells expression of CDKI p21 was higher than in the other lines (Figure 7b). Increased phosphorylation of p44 and p42 ERKs was also seen in fswt//fs188 versus fs164/fs120 cells (Figure 7c). Constitutive activation of JAK/Stat3 is a feature of cancer cells where its major function is to promote proliferation and survival [34]. Phospho-Stat3 levels were consistently higher in fswt and fs188 cells, compared to fs164 and fs120 cells; pan-JAK inhibitor P6 blocked Stat3 phosphorylation in all cell lines (Figure 7d). A similar pattern of pStat3 was evident in sub-confluent as in post-confluent cultures (data not shown), which demonstrated that unlike other systems its expression was not density-dependent [35]. Stat3 phosphorylation was also up-regulated in fs188 tumours *in vivo* (Figure 7e). Although Stat3 signalling is primarily associated with proliferation and survival, previous studies have shown that sustained activation of Ras/Raf/ERK led to the production of leukemia inhibitory factor (LIF), which then signaled through JAK/Stat3 in an autocrine or paracrine manner to mediate cell cycle arrest [36]. Figure 7f shows that fs164 and fs120 cultures produced significantly more secreted LIF than fswt and fs188 cells. It is therefore unlikely that signalling through LIF was responsible for the differences in levels of active JAK/Stat3 that were seen between the cell lines.

### Fibrosarcoma cells express tyrosine kinase VEGF receptors, which cannot be activated by VEGF

**Figure 8:**
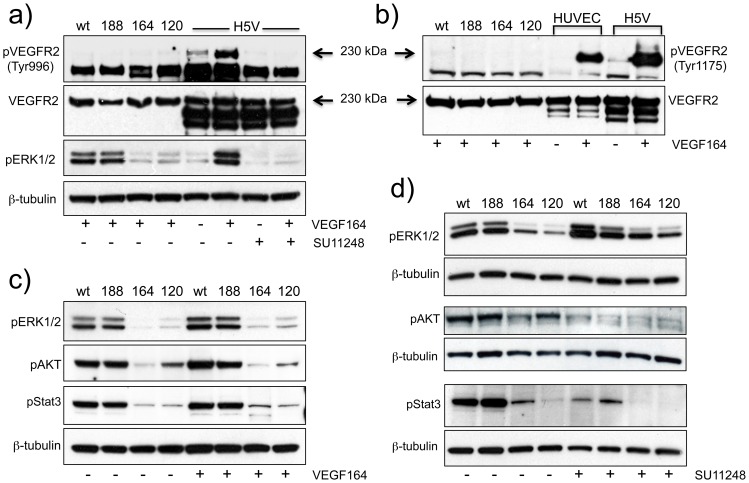
Analysis of VEGFR2 expression/activation in fibrosarcoma cells. **a,b,c**): Fibrosarcoma cells, HUVEC or H5V endothelial cells were stimulated with 100 ng/ml recombinant VEGF164 for 10 min. Equal amounts of proteins (10–30 µg) were analysed for **a**): Phospho-VEGFR2 (tyr996), total VEGFR2 and pERK1/2. SU11248 (10 µM) was added 5 min prior to VEGF; **b**): phospho-VEGFR2 (tyr1175) and total VEGFR2±10 µM SU11248; **c**): p-ERK1/2, pAKT and pStat3. **d**): Cells were incubated with SU11248 (10 µM) for 1 h before proteins were extracted and analysed for pERK1/2, pAKT and p-Stat3 (p-Stat3). All blots were normalized with an antibody to β-tubulin and are representative of at least three independent experiments.

We next explored the possibility that the observed differences in growth and survival-associated signalling were due to VEGF isoform-dependent autocrine actions. All fibrosarcoma cells expressed similar levels of VEGF receptor 2 (VEGFR2/flk-1) (Figure 8a) which could potentially mediate autocrine signalling. However, VEGFR2 was not constitutively phosphorylated/activated in any of the cells (not shown), and furthermore, exogenously added recombinant murine VEGF164 failed to activate its phosphorylation either at tyrosine 996 (Figure 8a) or at tyrosine 1175 (Figure 8b). Downstream ERK1/2, AKT and Stat3 were also not further activated/phosphorylated beyond basal levels by recombinant VEGF, results that were consistent with the failure of VEGF to activate its receptor(s) (Figure 8c). In parallel, VEGF induced robust VEGFR2 and ERK1/2 phosphorylation in HUVEC and H5V mouse endothelial cells (Figure 8a,b). In addition to VEGF164, other recombinant VEGF isoforms (VEGF120, VEGF188 and VEGF165) were tested over a range of concentrations (1, 10 and 100 ng/ml) but in all cases induction of VEGFR2 and/or ERK1/2 phosphorylation was not detected (data not shown). The VEGF receptor tyrosine kinase inhibitor SU11248 [37] blocked both basal and VEGF-induced VEGFR2 and pERK phosphorylation in H5V cells (Figure 8a) and HUVEC (data not shown) but interestingly had no effect on basal levels of pERK1/2 in the fibrosarcomas (Figure 8d). These results suggest that although the fibrosarcoma cells express VEGFR2, this nevertheless remains inactive/inactivatable. Furthermore, these data strongly indicate that the markedly higher levels of constitutively active pERK1/2, which were evident in fswt/fs188 compared to fs164/fs120 cells were unlikely to have arisen through VEGF-dependent autocrine activity through tyrosine kinase VEGF receptors. However, SU11248 caused a significant reduction in constitutive levels of AKT and Stat3 phosphorylation of in all the fibrosarcoma cells (Figure 8d); after overnight incubation with SU11248, the phosphorylation levels of both AKT and Stat3 returned back to their original basal levels (data not shown).

The fibrosarcoma cells also expressed full length VEGFR1/flt1 as well as a truncated variant most likely consisting of the intracellular domain of the receptor (see supplementary data, Figure S2) previously described in endothelial and breast cancer cells [38]. The truncated variant was constitutively phosphorylated at tyr1333 in the fibrosarcomas cells but this phosphorylation could not be downregulated by SU11248. Phosphorylated VEGFR1 was also evident in H5V cells; in these cells SU11248 caused a small reduction in VEGFR1 phosphorylation (see Figure S2).

## Discussion

**Figure 9:**
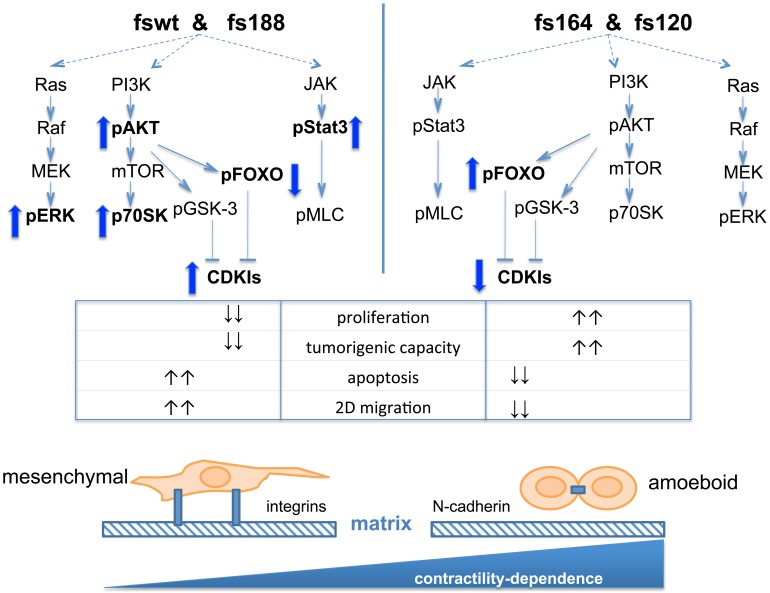
Schematic of proposed signalling interactions and morphological and motility characteristics of fibrosarcomas expressing single VEGF isoforms. Differences in PI3K/AKT, Ras/Raf/MEK/ERK and JAK/Stat3 signalling between fswt/fs188 and fs164/fs120 cells are shown. Upregulation/downregulation of activities or levels of expression of individual proteins are indicated by up or down arrows. VEGF188 expression is associated with reduced proliferation rates, increased apoptosis and typical integrin-dependent mesenchymal morphology and migration mode. VEGF164/VEGF120 expression is associated with faster proliferation rates, increased survival and rounded/amoeboid integrin-independent morphology and mode of motility. Migration of fs164/fs120 but not fswt/fs188 cells is contractility-dependent.

In this study, we demonstrate that endogenous single VEGF isoform expression is linked to distinct growth, survival and migratory characteristics in Ras transformed fibrosarcoma cells. Our model system allowed us to investigate three individual isoforms of murine VEGF (VEGF188, VEGF164 and VEGF120), when expressed under the control of the gene's physiological promoter, and hence has a distinct advantage compared to over-expression systems that may alter normal endogenous signalling interactions. We show that VEGF188 expression is linked to a slower rate of tumour cell proliferation and decreased survival. Furthermore, VEGF188 expression was associated with strong cell-matrix matrix interactions and a mesenchymal morphology and mode of cell motility *in vitro*. In contrast, VEGF164 and VEGF120 endogenous expression was associated with more rapid cell proliferation, increased survival and a rounded amoeboid morphology and mode of motility *in vitro* (summarised in Figure 9). Differences between the various cell phenotypes are very unlikely to have arisen by clonal selection during the generation of the cell lines, as several individual clones isolated after transformation maintained identical properties to their corresponding parental lines (data not shown).

The cells we developed are of mesenchymal origin and accordingly they all constitutively expressed classic mesenchymal markers such as N-cadherin (Figure 3c), alpha smooth muscle actin and vimentin (unpublished data). However, despite not displaying typical mesenchymal features, in terms of their morphology and migratory characteristics, the fs164 and fs120 cells express higher levels of N-cadherin compared to the more typically mesenchymal fswt and fs188 cells. We postulate that the higher levels of N-cadherin protein facilitate and sustain the distinct cell-cell contacts that fs164 and fs120 cells form in 2D culture. Indeed, previously it was shown that upregulated N-cadherin expression mediated cell-cell adhesion by localising to cell-cell contacts in invasive epithelial MDCK cells after undergoing epithelial-to-mesenchymal transition (EMT) [39].

Differences in adhesion to various matrix proteins are also suggestive of differences in integrin expression and/or status of activation. These differences are likely to involve both alpha and beta integrin subunits. While all fibrosarcomas expressed integrin β1, fs188 cells expressed higher levels of ILK. ILK stabilises β1 adhesions, is associated with integrin engagement/activation and was found to be over-expressed in mesenchymal cancer cells [40], [41]. Therefore, upregulated ILK expression could potentially contribute to a more adhesive mesenchymal fs188 phenotype. Amoeboid morphology, which is less dependent on integrin-matrix interactions, is more readily seen in culture when cells are grown on fibrillar collagen matrices. Indeed, fs164 and to a large extent fs120 cells were rounded on collagen (Figure 2a). Interestingly, fs164 and fs120 cells have rounded cell morphologies when grown as subcutaneous tumours *in vivo* and fs188 tumours retain an elongated mesenchymal cellular morphology (see Figure 4c).

The extent of anchorage independent growth in agar was consistent with growth on solid surfaces. Growth in agar reflects tumorigenic potential and results *in vitro* correlate with our previous findings *in vivo*, where expression of VEGF164 and VEGF120 was associated with a more rapid initiation of tumour growth [14].

Although VEGF is a pro-survival factor, there are suggestions that VEGF188 may elicit pro-apoptotic signals as shown in Figure 4. Chondrocytes from homozygous VEGF188/188 mice apoptosed at high rates during development, and could be rescued by exogenous VEGF164 [42]. In addition, over-expression of VEGF189 in breast cancer cells induced apoptosis via NRP-1 [43]. Interestingly, the fswt and fs188 cells markedly over-express NRP1 compared to the other two cell lines (see Figure S2d). It is therefore possible that NRP-1 may regulate pro-apoptotic processes associated with VEGF188 expression in the fibrosarcomas. Our results are therefore in agreement with the above studies and suggest that increased pro-apoptotic signalling is an intrinsic property of VEGF188 expression. It was, however, intriguing that the pro-survival and pro-proliferative signalling proteins AKT, ERK1/2 and Stat3, were more highly activated in VEGF188-expressing cells. A potential explanation for this discrepancy came from the analysis of downstream PI3K/AKT effectors. While AKT target p70S6 was phosphorylated, unexpectedly, FOXO1, another key target of AKT and regulator of cell cycle and pro-survival signalling [44], was more highly phosphorylated in fs164 and fs120 cells (Figure 7b). Inactive/phosphorylated FOXO1 correlated with lower levels of p21 and p27 in fs164 and fs120 cells. In contrast, in fs188 cells, p27 was markedly upregulated and in wild type cells, p21 was more highly expressed, thus correlating with their slower proliferation rates. The mechanism(s) through which active AKT signalling couples to p70S6 but not FOXO and GSK-3 in fs188 remains unclear. PP2A phosphatase regulates the activity of FOXO1 and GSK-3 [45], [46]. It is possible that high phosphatase activity dephosphorylates FOXO and GSK3 in fs188 cells. Several studies have suggested that the pro-survival function of AKT can be overridden [47]. Chronic AKT activation can contribute to apoptosis induction, while many stress-inducing agents and chemotherapeutic drugs activate AKT. Indeed, pro-apoptotic agents often activate survival signals, including Stat3 and ERKs in addition to AKT, as part of a protective response [48]. The fact that Stat3 was highly active in both fs188 cells and solid tumours *in vivo* suggest that high levels of Stat3 activity represent an intrinsic property consequent to VEGF188 expression.

High levels of constitutively phosphorylated AKT and presence of lamellipodia suggests a link between the PI3K/AKT pathway and the mesenchymal mode of migration of fswt and fs188 cells. Our results are in agreement with studies showing that mesenchymal cell lines exhibit high levels of AKT phosphorylation [41], [49]. PI3K/AKT signalling is also a critical component of the epithelial-mesenchymal transition [50], [51]. Ras-mediated activation of ERKs promotes activation of Rac and lamellipodia formation to drive tumour cell motility [52]. Our results are therefore in line with these studies and suggest that the features of cells expressing VEGF-188 are linked with activated Ras/PI3K and Ras/ERK.

Differential requirement for integrin engagement and matrix adhesion as well as actinomyosin contractility are major features that distinguish amoeboid from mesenchymal migration [20], [53], [54]. In mesenchymal cells, Rho/ROCK driven contractility supports the formation of focal adhesions and contractile stress fibers that can generate traction force for forward movement, while Rac and Cd42 are required for the extension of protrusions at the cell's leading front [28], [55]. Mesenchymal movement is also dependent on integrin-matrix interactions. On the other hand, in rounded amoeboid cells integrin-matrix adhesive interactions are weak, while cortical myosin II generated contraction is essential for migration in 3D matrices [53]. Several studies have shown that loss of contractility switches cells over to a mesenchymal mode of movement [30], [54], [55]. Inhibitors of Rho/ROCK or contractility impaired amoeboid movement but had minimal effects on migration in mesenchymal tumour cells thus illustrating that Rho/ROCK signalling/contractility are essential in amoeboid but dispensable in mesenchymal motility [30], [54], [55]. When exposed to contractility inhibitors, the fs164 and fs120 cells acquired mesenchymal characteristics (they spread more and formed more protrusions) and were able to migrate faster on 2D surfaces. Our results are in agreement with the studies described above in that inhibitors of contractility had no significant effect on fswt or fs188 cells but altered the morphology and motility of fs164 and fs120 cells. In our study, all the fibrosarcomas expressed similar levels of phosphorylated MLC, a read-out of actinomyosin contractility (Figure 6c), suggesting that contractility alone is not sufficient to dictate morphology and migration. Inhibition of Jak/Stat3 signalling abrogated the phosphorylation of MLC as described previously for melanoma cells [30] thus establishing this pathway as a major driver of contractility also in fibrosarcomas. However, while in melanoma cells, pStat3 correlated with amoeboid mode of movement, in our system pStat3 expression correlated with a mesenchymal morphology that was independent of contractility for migration. Others have shown that mesenchymal hepatocellular cancer cells expressed higher levels of pStat3 compared to their epithelial counterparts [41]. Interestingly, inhibition of JAK signalling resulted in a marked upregulation of integrin β1 expression, which correlated with a switch to a more mesenchymal morphology, particularly in fs164 and fs120 cells.

We hypothesised that the observed differences in the signalling characteristics of the fibrosarcoma cells could have arisen through isoform-dependent autocrine activity, mediated by tyrosine kinase VEGF receptors. However, although the fibrosarcoma cells expressed RTK VEGF receptors, these could not be activated/phosphorylated by recombinant VEGF isoforms. Exogenously added VEGF also failed to activate downstream signalling to pERK1/2, pAKT and pStat3 in the fibrosarcoma cells, in marked contrast to its robust activation of VEGF signalling in endothelial cells. Furthermore, adding back different recombinant VEGF isoforms had no effect on fibrosarcoma cell proliferation (supplementary Figure S3) and had no influence on cell morphology (data not shown). Sunitinib (SU11248) a potent RTK inhibitor with VEGFR selectivity [37] had no effect on basal pERK1/2 levels further confirming that the intrinsic pERK1/2 levels were VEGFR-independent. Sunitinib did inhibit pAKT and pStat3 but this is most likely due to inhibition of other RTK targets of sunitinib, such as PDGFRβ. Previously it was shown that VEGF produced by renal carcinoma cells promoted tumour cell growth through activating NRP-1 receptors and downstream Ras [7]. While our results cannot exclude an involvement of NRP-1 in autocrine signalling, the fact that various recombinant VEGF isoforms failed to activate pERK, pAKT or pStat3 signalling or alter the proliferation of the fibrosarcoma cells does not support such an involvement.

Taken together our data suggest that the inherent differences in signalling, proliferation, survival and migration between our different cell lines are a result of complex interactions governed by endogenous VEGF isoform expression, that are independent of simple ligand-receptor activation. VEGF 189 has been shown to localise to the nucleus [56], [57], which could be significant here. Although the function of VEGF in the nucleus has not been established, nuclear localised VEGFD was found associated with c-myc and RNA polymerase II and regulated c-myc-dependent gene transcription in fibroblasts in a VEGF receptor-independent manner [58]. It is therefore possible that VEGFA isoforms also have intracrine activity that is not dependent on classic receptor activation.

In summary, we present, for the first time, evidence that VEGF188 expressed by tumour cells is associated with mesenchymal motility, slower proliferation, and increased apoptosis, while VEGF164 and VEGF120 are associated with a more rounded/amoeboid morphology and increased proliferation and survival. Both amoeboid and mesenchymal modes of movement are important in metastasis and different targeting approaches may be needed to attack different invasive strategies. In this respect, it is of interest that VEGF189 isoform over-expression was shown to correlate with metastasis and poor prognosis in colon and lung cancer [59], [60]. Deciphering the various processes by which individual VEGF isoforms contribute to tumour growth is important for the design of cancer therapies. Until now, such therapies focused on targeting VEGF signalling with an aim to impair angiogenesis. The demonstration that other non-angiogenesis dependent aspects of tumour growth are reliant on VEGF isoform expression adds to the complexity of VEGF signalling within the tumour microenvironment and necessitates a better understanding of the molecular mechanisms involved.

## Supporting Information

Figure S1 **Growth of fibrosarcoma cells in soft agar.** Colonies formed by fibrosarcoma cells grown in soft agar and imaged using a 10× objective.
(TIF)

Figure S2 **VEGFR1 and NRP-1 expression by fibrosarcoma cells.** **a**) Schematic diagram showing VEGFR1 domains recognised by two different commercial VEGFR1 antibodies (Abcam). Antibody ab32152 was raised against a synthetic peptide corresponding to residues in the N-terminal extracellular domain of VEGFR1 and antibody ab2350 was raised against a synthetic peptide to C-terminal residues, within the tyrosine kinase domain of the receptor; **b**) ab32152 recognized the full length receptor (180 kDa) in fibrosarcoma cells while antibody ab2350 recognised the full-length receptor as well as a truncated variant (120 kDa) most likely corresponding to the receptor intracellular domain. The truncated variant was also present in endothelial cells. **c**) The truncated variant was constitutively phosphorylated at tyr1333 in both fibrosarcoma and H5V cells but its phosphorylation could not be blocked by SU11284. d) NRP-1 expression in the fibrosarcomas.
(TIF)

Figure S3 **Fibrosarcoma cell proliferation in the presence of recombinant VEGF isoforms.** Cells were plated in 6-well plates at a density of 2×10^4^ cells per well for and treated with the indicated amounts of recombinant VEGF isoforms. **a**) fs164 cells were treated with rVEGF164 or rVEGF188; b) fs120 cells were treated with rVEGF120 or rVEGF188; **a,b**) Cells were counted after 5 days in culture. Results (cell counts ±SD) are from one of two repeat experiments.
(TIF)
