# Supplementary figures and images for: Tumour Cells Expressing Single VEGF Isoforms Display Distinct Growth, Survival and Migration Characteristics
Source: PLoS One. 2014 Aug 13;9(8):e104015. doi: 10.1371/journal.pone.0104015 (PMC4131915; doi:10.1371/journal.pone.0104015)

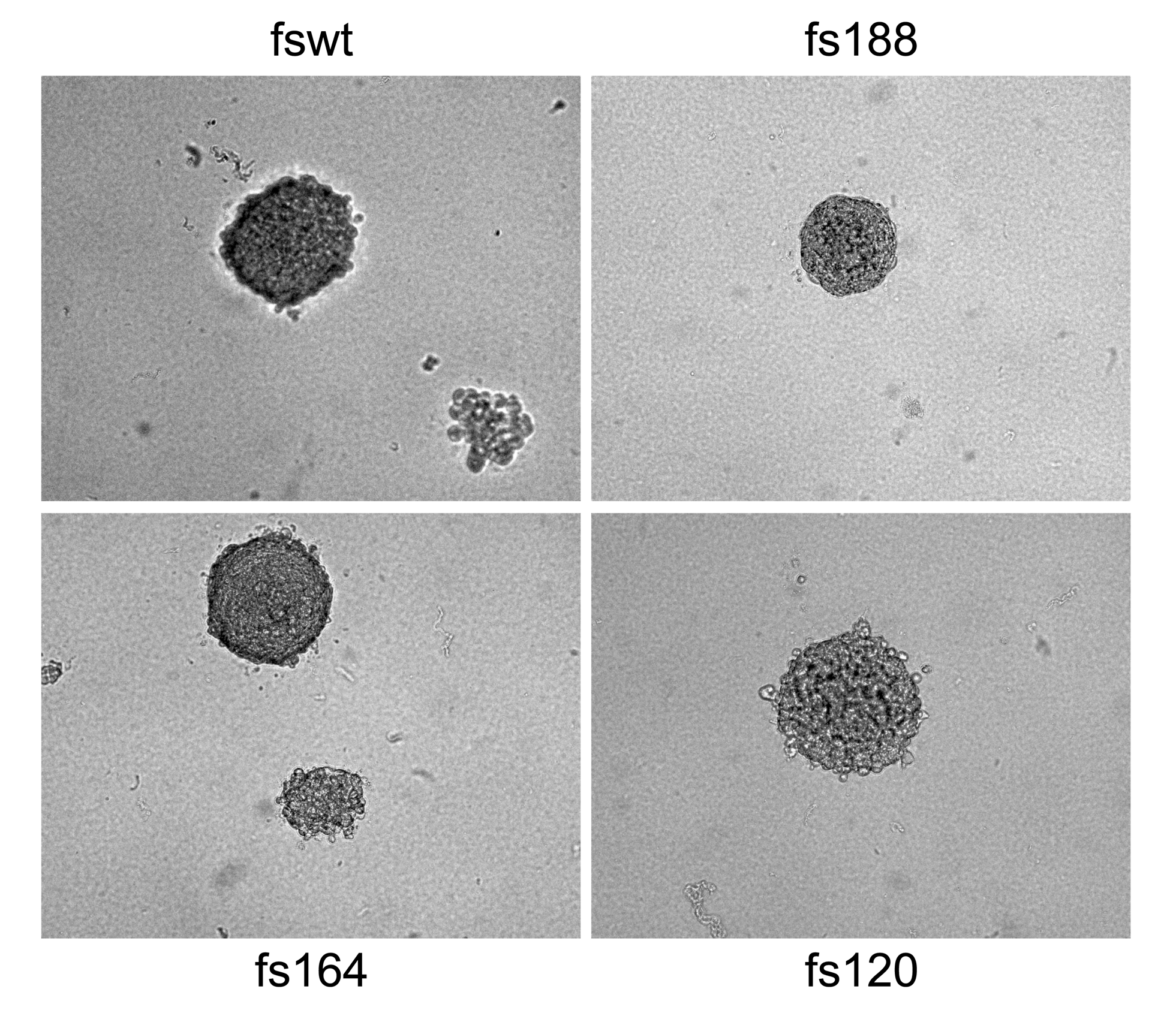

Supplement: Figure S1 — Growth of fibrosarcoma cells in soft agar. Colonies formed by fibrosarcoma cells grown in soft agar and imaged using a 10× objective. (TIF) [file pone.0104015.s001.tif]

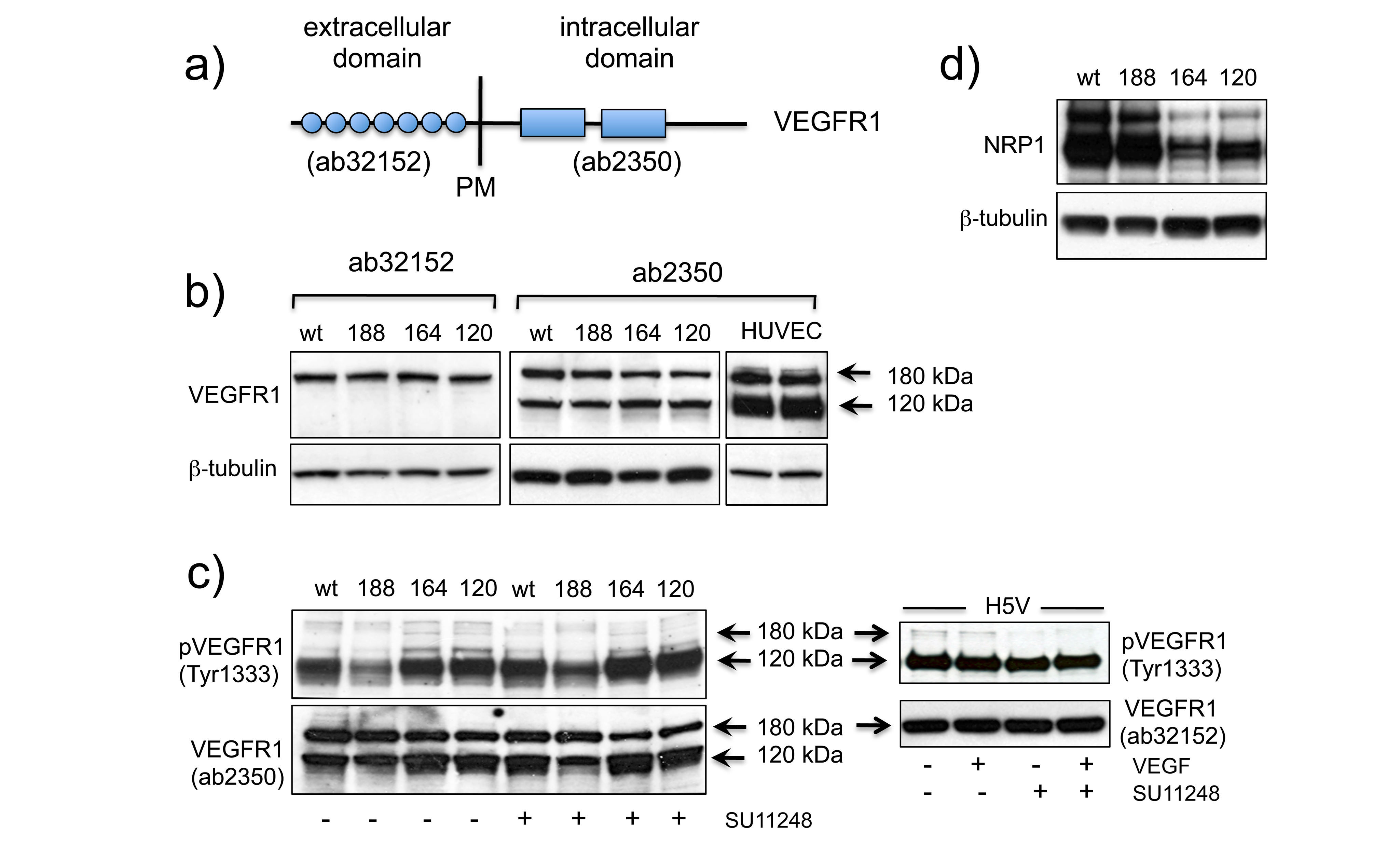

Supplement: Figure S2 — VEGFR1 and NRP-1 expression by fibrosarcoma cells. a) Schematic diagram showing VEGFR1 domains recognised by two different commercial VEGFR1 antibodies (Abcam). Antibody ab32152 was raised against a synthetic peptide corresponding to residues in the N-terminal extracellular domain of VEGFR1 and antibody ab2350 was raised against a synthetic peptide to C-terminal residues, within the tyrosine kinase domain of the receptor; b) ab32152 recognized the full length receptor (180 kDa) in fibrosarcoma cells while antibody ab2350 recognised the full-length receptor as well as a truncated variant (120 kDa) most likely corresponding to the receptor intracellular domain. The truncated variant was also present in endothelial cells. c) The truncated variant was constitutively phosphorylated at tyr1333 in both fibrosarcoma and H5V cells but its phosphorylation could not be blocked by SU11284. d) NRP-1 expression in the fibrosarcomas. (TIF) [file pone.0104015.s002.tif]

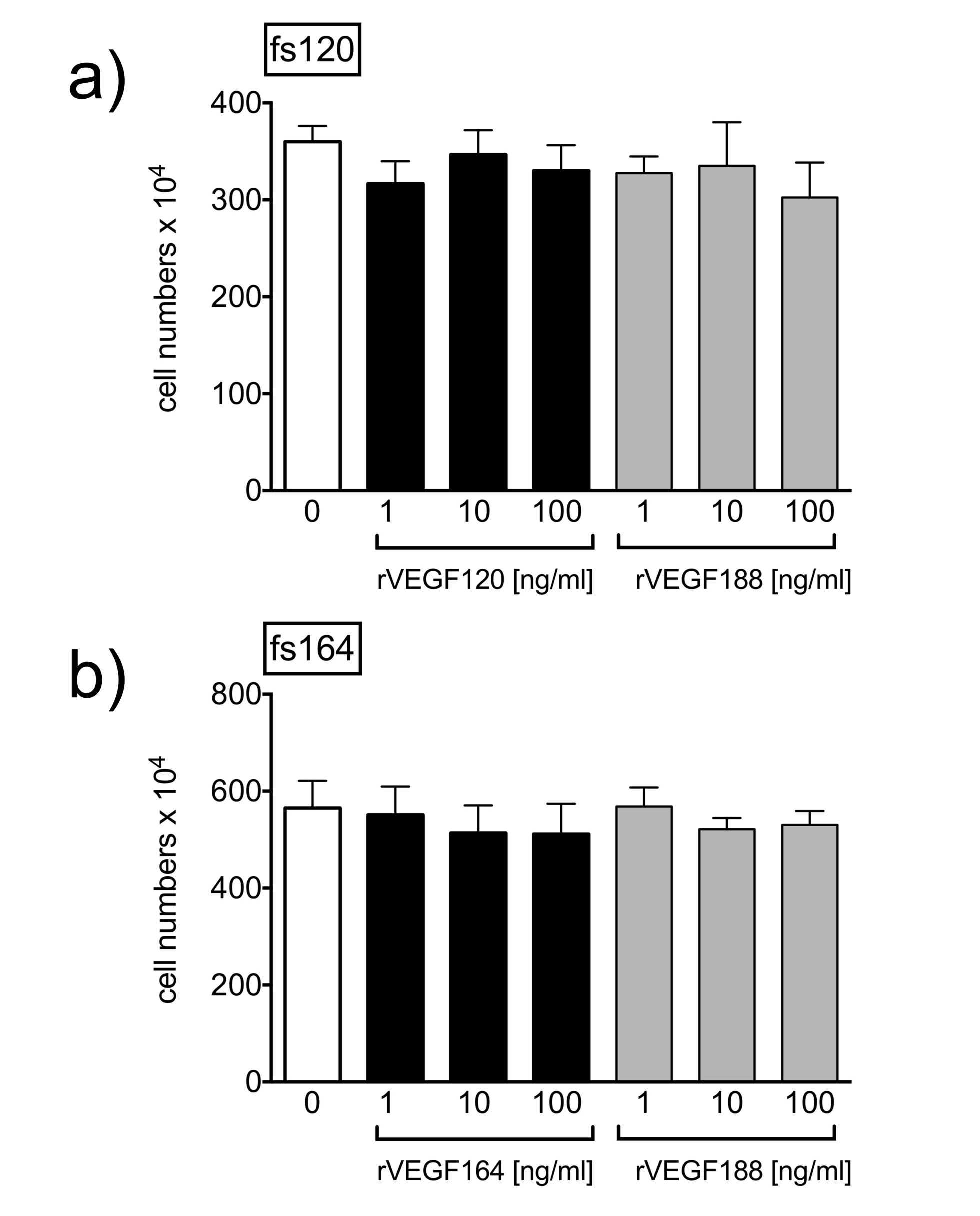

Supplement: Figure S3 — Fibrosarcoma cell proliferation in the presence of recombinant VEGF isoforms. Cells were plated in 6-well plates at a density of 2×10^4^ cells per well for and treated with the indicated amounts of recombinant VEGF isoforms. a) fs164 cells were treated with rVEGF164 or rVEGF188; b) fs120 cells were treated with rVEGF120 or rVEGF188; a,b) Cells were counted after 5 days in culture. Results (cell counts ±SD) are from one of two repeat experiments. (TIF) [file pone.0104015.s003.tif]
